# Temporal Trends and Mortality of Vancomycin-Resistant *Enterococcus* Bacteremia—A Six-Year Retrospective Cohort Study in a Tertiary Hospital in Greece

**DOI:** 10.3390/pathogens15050467

**Published:** 2026-04-25

**Authors:** Despoina Kypraiou, Angelos Sourris, Eirini Astrinaki, Efsevia Vitsaxaki, Stamatina Saplamidou, Maria Vakonaki, Kyriaki Tryfinopoulou, Georgios Chamilos, Petros Ioannou, Diamantis Kofteridis

**Affiliations:** 1 School of Medicine, University of Crete, 71003 Heraklion, Greece; 2 Internal Medicine Department, University Hospital of Heraklion, 71110 Heraklion, Greece; 3 Infection Control Committee, University Hospital of Heraklion, 71110 Heraklion, Greece; 4 Clinical Microbiology Laboratory, University Hospital of Heraklion, 71110 Heraklion, Greece

**Keywords:** infection, *Enterococcus*, vancomycin-resistant *Enterococcus*, bacteremia, antimicrobial resistance, mortality

## Abstract

Background: Vancomycin-resistant *Enterococcus* (VRE) bacteremia represents a major therapeutic and epidemiological challenge, particularly in regions with high antimicrobial resistance rates such as Southern Europe. Longitudinal local data are essential to guide infection control and antimicrobial stewardship strategies. This study aimed to evaluate temporal trends in incidence, management, and mortality of VRE bacteremia in a tertiary care center in Greece over a six-year period, including comparison before and after the coronavirus disease 2019 (COVID-19) pandemic. Methods: This retrospective observational study included adult patients with VRE bacteremia at the University Hospital of Heraklion, Greece, from 2018 to 2023. Demographic and clinical data, such as the Pitt Bacteremia Index (PBI), as well as microbiological, and treatment data were collected from patient records. Incidence was calculated per 10,000 patient-days. Comparisons were performed between survivors and non-survivors and between pre- and post-COVID-19 eras. Multivariate regression analysis was used to identify predictors of in-hospital mortality. Results: A total of 96 patients were included (mean age 68.6 ± 14.5 years; 56.3% male). The incidence of VRE bacteremia increased more than five-fold during the study period, from 0.242 cases per 10,000 patient-days in 2018 to a peak of 1.344 per 10,000 patient-days in 2022, remaining elevated in 2023 (1.001 per 10,000 patient-days). The overall in-hospital mortality was 54.2%. Non-survivors had significantly higher PBI scores compared to survivors (median 2.5 vs. 0, *p* = 0.005). In the multivariate analysis, higher PBI was independently associated with in-hospital mortality [odds ratio: 1.449 (95% confidence intervals: 1.166–1.801)]. Appropriate empirical therapy was administered in 41.7% of cases and was not significantly associated with survival. Post-COVID-19 patients were older (69.9 vs. 61.4 years, *p* = 0.0365), and antimicrobial regimens were more frequently adjusted according to susceptibility testing (55.7% vs. 18.2%, *p* = 0.0141), but mortality did not significantly differ between periods. Conclusion: VRE bacteremia incidence increased dramatically over the six-year study period in our tertiary center, with persistently high mortality exceeding 50%. Severity of illness at the diagnosis of bacteremia, as measured by the PBI, was an independent predictor of in-hospital mortality. Strengthened infection prevention measures, optimized antimicrobial stewardship, and early aggressive management are urgently needed to mitigate the growing burden of VRE bacteremia.

## 1. Introduction

The genus *Enterococcus*, once considered a relatively low-virulence organism of the gastrointestinal tract, has evolved into an important cause of healthcare-associated infections (HAIs) globally [1,2]. Among these, *Enterococcus faecalis* and *Enterococcus faecium* are the most clinically significant species, accounting for a vast majority of human infections [3,4]. Over the last three decades, the therapeutic landscape for these pathogens has changed considerably with the emergence and rapid dissemination of Vancomycin-Resistant *Enterococcus* (VRE) [5].

VRE represents a clinical challenge due to limited therapeutic options and a significant public health threat, leading the World Health Organization (WHO) to categorize it as a “priority pathogen” for which new treatments are urgently needed [6]. The transition of enterococci from commensal organisms to multi-drug resistant (MDR) pathogens is associated with acquisition of resistance to glycopeptides, primarily mediated by the vanA and vanB gene clusters [7,8]. This allows these organisms to survive in the presence of vancomycin. This resistance is frequently found on mobile genetic elements, such as plasmids and transposons, facilitating horizontal gene transfer between species and even across different genera [7].

In clinical settings, VRE bacteremia is of high clinical importance. Bloodstream infections (BSIs) with VRE are associated with significantly higher morbidity and mortality compared to their vancomycin-susceptible counterparts [9]. The limited choices of effective antimicrobials, often restricted to linezolid and daptomycin, may lead to delays in appropriate empirical therapy, a factor that has been traditionally linked to poor clinical outcomes [10].

The management of VRE bacteremia is complicated by the patient populations it typically affects. Such patients often have significant underlying comorbidities, such as solid malignancies, diabetes mellitus, and chronic kidney disease, leading to a high likelihood of transition from colonization to invasive infection. Furthermore, the presence of indwelling medical devices is a primary driver of these infections [10,11].

Southern Europe, and Greece in particular, has consistently reported some of the highest rates of antimicrobial resistance (AMR) within the European Union [12]. Factors contributing to this include high levels of community antibiotic consumption and challenges in hospital infection control practices. In Greek tertiary centers, the pressure from MDR organisms often forces clinicians to use broad-spectrum agents empirically, further driving the selection of resistant strains such as VRE [13,14].

While the general trends of VRE are well-documented, local longitudinal data are essential for tailoring hospital-specific antimicrobial stewardship programs and infection control policies. This study seeks to analyze the temporal trends in the management and mortality of VRE bacteremia at the University Hospital of Heraklion, Greece, over a six-year period.

## 2. Materials and Methods

This observational retrospective study was conducted over a 6-year period from 2018 to 2023 at the University Hospital of Heraklion, in Crete, Greece. The study included adult patients (18 years and above) with bacteremia caused by vancomycin-resistant *Enterococcus* species. Patients under the age of 18 years old were excluded. The diagnosis of VRE bacteremia was defined as the isolation of VRE from at least one blood culture that was considered by clinicians to be a cause of infection due to the presence of symptoms and signs of infection, such as fever, tachycardia, increased respiratory rate, hemodynamic instability, leukocytosis or leukopenia, or increased inflammatory markers. Patients’ data were collected retrospectively from the hospital information system by the study’s investigators using a standardized data collection form spanning different categories such as demographic information of the patient (age, gender) and the type of infection, setting of infection acquisition (nosocomial or community), antimicrobial therapy, and antimicrobial susceptibility. In patients with more than one episode, only the first episode was included in the study. The infection was considered nosocomial if the onset of bacteremia occurred at least 48 h after admission. Additionally, data regarding risk factors for infection (presence of indwelling devices, immunosuppression, prior antibiotic exposure within 30 days) and comorbidities (diabetes mellitus, cancer, hematological malignancy and chronic kidney diseases), as well as calculation of Charlson Comorbidity Index (CCI), were collected. The Pitt bacteremia index (PBI) was calculated for each episode of bacteremia [15]. Polymicrobial BSIs were defined as infection with the identification of more than one pathogen isolated from blood cultures. The source of the bloodstream infection was evaluated using bacterial culture obtained from the presumed source and medical chart review of the clinical examination and assessment of the treating clinician and/or the infectious disease specialist who consulted. Primary bacteremia was defined as bacteremia of no identifiable source.

Blood culture bottles were incubated using the BACT/ALERT^®^ VIRTUO (BioMerieux, Marcy, l’Etoile, France), a fully automated blood culture system. Positive blood cultures were processed as per the standard operating procedures of the clinical microbiology laboratory. Species identification was performed by matrix-assisted laser desorption/ionization mass spectrometry (MALDI-TOF MS) on isolated colonies (VITEK-MS, BioMerieux), and susceptibility testing using the VITEK-2 automated system (BioMerieux). The respective EUCAST clinical breakpoints for the years 2018–2023 were used.

Descriptive statistics were performed with GraphPad Prism 6.0 (GraphPad Software, Inc., San Diego, CA, USA). Categorical data were analyzed with Fisher’s exact test. Continuous variables were compared using the Mann–Whitney U-test for non-normally distributed variables or t-test for normally distributed variables. All tests were two-tailed and a *p*-value equal to or lower than 0.05 was considered significant. Data are presented as numbers (%) for categorical variables and medians [interquartile range (IQR)] for continuous variables. All parameters were calculated with GraphPad Prism 6.0 (GraphPad Software, Inc., San Diego, CA, USA). A multivariate logistic regression analysis of in-hospital mortality was performed with the factors identified to have a *p*-value of less than 0.1 in the comparison between patients who survived versus those who died. Multivariate analysis was performed using the SPSS version 23.0 (IBM Corp., Armonk, NY, USA).

The study was conducted in accordance with the Declaration of Helsinki, and approved by the Institutional Review Board of the University Hospital of Heraklion (protocol number 7/05-03-2025, 5 March 2025).

## 3. Results

### 3.1. Demographic and Baseline Clinical Characteristics

The study cohort comprised 96 adult patients diagnosed with VRE bacteremia at the University Hospital of Heraklion between 2018 and 2023. Only three episodes were diagnosed within 48 h after admission and, thus, were not nosocomial. The demographic profile of the participants revealed a slight male predominance, with 54 individuals (56.3%) being men. The overall mean age of the cohort was 68.6 years [Standard Deviation (SD) ± 14.5]. Clinical assessment at the time of infection indicated a significant burden of underlying disease, as reflected by a median CCI of 5 [Interquartile Range (IQR) 3–7]. Specific comorbidities were prevalent throughout the population, most notably: solid malignancy, which was identified in 28.6% (26/91) of the patients, diabetes mellitus, affecting 26.4% (24/91) of the study group, myocardial infarction, recorded in 15.4% (14/91) of cases, chronic lung disease and heart failure, both present in 13.2% (12/91) of the population, hematologic malignancy, which was diagnosed in 9.9% (9/91) of the patients and cerebrovascular disease, noted in 8.8% (8/91) of the cohort. Furthermore, a high proportion of the patients (81.3%, *n* = 78) were utilizing a central venous catheter at the onset of their bacteremia. Among all episodes of VRE bacteremia, 45 (46.9%) were diagnosed in medical departments, 39 (40.6%) in the intensive care unit (ICU), and 12 (12.5%) in surgical departments. All episodes were caused by *Enterococcus faecium* except from one that was caused by *Enterococcus faecalis*.

In general, infection occurred after a median of 20 days of stay in the hospital. Primary bacteremia, defined as having no identifiable source, was the most common presentation, occurring in 56.8% (54/95) of the total cohort. Polymicrobial infections were also frequent, identified in 41.7% (*n* = 40) of cases. The severity of the illness at the time of the first positive blood culture, as measured by the PBI, had a median value of 2 (IQR 0–4). Appropriate empirical treatment was administered to 41.7% (35/84) of patients, most commonly daptomycin in 85.7% of them (30), and linezolid in 14.3% (5). The most commonly used antimicrobials in the case of inappropriate empirical treatment were vancomycin in 49% (24) and tigecycline in 18.4% (9) of patients treated inappropriately. A change in antimicrobial therapy was performed in 35 patients among 49 that were treated with an inappropriate empirical antimicrobial. The most commonly used antimicrobials in targeted therapy were linezolid in 51.4% (18 patients) and daptomycin in 42.9% (15). The median duration of appropriate treatment for the entire cohort was 9 days (IQR 4.8–14). Table 1 and Table 2 show the characteristics of patients with VRE bacteremia.

### 3.2. Comparative Analysis Between Survivors and Non-Survivors

Table 1 and Table 2 show the characteristics of patients with VRE bacteremia in total and with regard to whether they survived. When comparing survivors and non-survivors, no significant differences were observed in age, sex, or comorbidity burden, although non-survivors had a trend toward higher CCI (median 5 vs. 4, *p* = 0.0656). The PBI was calculated for every patient in the present study, in an effort to calculate the severity of the disease. The results showed that non-survivors had a higher median PBI, 2.5 (IQR 0–6), in contrast to 0 (0–2) among the survivors, with a *p*-value of 0.0051. Patients who were in a surgical ward when diagnosed with VRE bacteremia were more likely to survive.

Follow-up blood cultures were performed less frequently in non-survivors compared to survivors (76.5% vs. 97.7%, *p* = 0.0027). In addition, in the survivors’ group, longer duration of appropriate antimicrobial therapy was provided in contrast to the patients who ultimately died (median 12 vs. 7 days, *p* = 0.0115). Survivors had a longer duration of hospitalization (median 25 vs. 10.5 days, *p* = 0.0009).

### 3.3. Yearly Trends and Incidence Analysis

**Figure 1:**
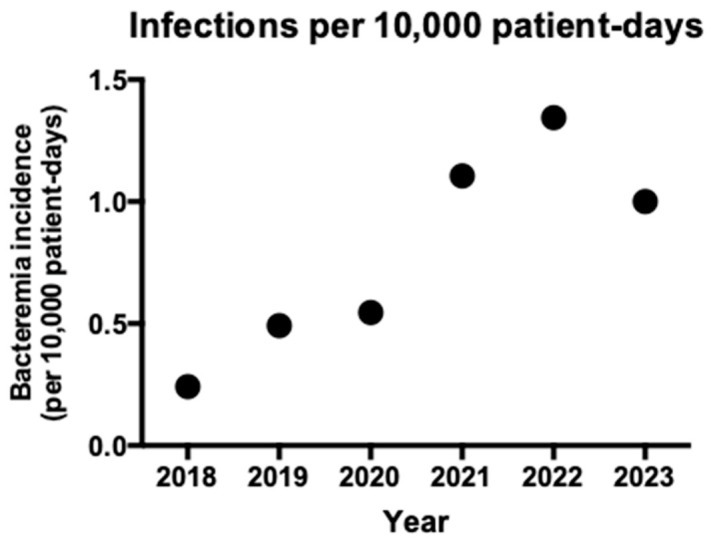
Incidence of vancomycin-resistant *Enterococcus* bacteremia in the timeframe of the study.

The incidence of VRE bacteremia showed a dramatic and sustained increase throughout the six-year observation period. Total patient-days remained relatively stable, ranging from a low of 183,272 in 2020 to a high of 217,726 in 2023. However, the frequency of VRE cases per 10,000 patient-days surged significantly; in 2018, the incidence was 0.242, with only 5 cases recorded. By 2021, this figure had more than quadrupled to 1.106 per 10,000 patient-days (*n* = 22). The peak was reached in 2022, with an incidence of 1.344 (*n* = 28), representing more than a five-fold increase from the start of the study. In 2023, the incidence remained elevated at 1.001 (*n* = 21). Table 3 and Table 4 show the characteristics of patients with VRE bacteremia per year of the study period and Figure 1 shows the incidence of VRE bacteremia in the timeframe of the study.

### 3.4. Comparative Analysis: Pre-COVID-19 vs. Post-COVID-19 Eras

A focused analysis was conducted to compare the characteristics of patients before (*n* = 15) and after (*n* = 81) the onset of the coronavirus disease 2019 (COVID-19) pandemic. Tables S1 and S2 show the characteristics of patients with VRE bacteremia before and after the COVID-19 pandemic. There was a notable increase in the mean age of patients in the post-COVID-19 era (69.9 years, SD ± 13.3) compared to the pre-pandemic era (61.4 years, SD ± 18.9), with a *p*-value of 0.0365. In the post-COVID-19 period, clinicians were significantly more likely to change antimicrobial therapy based on susceptibility results (55.7% vs. 18.2%, *p* = 0.0141). Despite these changes, the in-hospital mortality rate did not differ statistically between the two periods (40% pre-COVID-19 vs. 56.8% post-COVID-19, *p* = 0.2684).

### 3.5. Predictors of In-Hospital Mortality

To evaluate and identify factors associated with in-hospital mortality, a multivariate logistic regression analysis was performed with factors previously identified to have a *p*-value less than 0.1 in the comparison of patients who survived compared to those who died with the exception of duration of appropriate treatment and the duration of hospitalization which are not expected to be independent variables of in-hospital mortality. The analysis identified only PBI to be independently positively associated with in-hospital mortality (*p*-value = 0.001). Table 5 shows the results of the multivariate regression analysis.

## 4. Discussion

In this six-year retrospective cohort study in a tertiary care center in Greece, a major finding was the marked and sustained increase in VRE bacteremia and a persistently high in-hospital mortality. More specifically, a more than five-fold rise in VRE bacteremia incidence during the study period was noted, along with an overall in-hospital mortality exceeding 50%, a high severity of illness, as shown by the PBI, that was identified to be directly independent predictor of mortality, and some temporal shifts in patient characteristics and management practices after the onset of the COVID-19 pandemic.

The most important finding of the present study is the dramatic increase in VRE bacteremia incidence, from 0.242 cases per 10,000 patient-days in 2018 to a peak of 1.344 in 2022, with persistently elevated levels in 2023. This rise occurred despite relatively stable annual patient-days, suggesting a true epidemiological shift rather than a denominator effect. These findings are in line with broader European trends indicating increasing rates of antimicrobial resistance, particularly in Southern Europe [16]. Greece has historically reported among the highest rates of antimicrobial consumption and multidrug-resistant organisms in the European Union [12]. The sustained increase observed in the present study may reflect cumulative antimicrobial pressure, ongoing challenges in infection prevention and control, and possible clonal dissemination within the hospital setting [17,18]. On the other hand, VRE bacteremia could, at least in some patients, present a breakthrough infection following treatment of Gram-negative MDR pathogens that are endemic in Greek hospitals [19,20,21]. To that end, it is of note that the median length of stay before the development of VRE bacteremia was 20 days, suggesting significant selective pressure of the patients’ microbiome, or a long time allowing for exposure and colonization by VRE. Although molecular typing was not performed, the magnitude and rapidity of the rise raise concerns about potential horizontal spread of high-risk VRE clones. Interestingly, a study in another hospital in Greece evaluating VRE bacteremias in the same six year period also noticed an increase in the incidence of bacteremia by these resistant pathogens [22].

The COVID-19 pandemic may have further amplified this increase in VRE bacteremia incidence. Increased empirical broad-spectrum antibiotic use, healthcare system strain, prolonged hospitalizations, and disruptions in infection control practices during pandemic surges may all have contributed to the acceleration observed after 2020 [23,24,25]. Although our pre- and post-COVID-19 comparison did not demonstrate a statistically significant difference in mortality, the post-pandemic period accounted for the vast majority of cases and was characterized by older patients and more frequent antimicrobial adjustments based on susceptibility results.

The overall in-hospital mortality rate of 54.2% noted in the present study highlights the poor prognosis associated with VRE bacteremia. The rates reported in the present study are at the higher end of mortality rates reported in the literature, which typically range from 20% to 50%, depending on the patient population and the severity of illness [26,27,28]. The high comorbidity burden of the present cohort that is implied by the high median CCI, the advanced patients’ age, and the high frequency of central venous catheter use may have contributed to these outcomes [29].

Importantly, among all evaluated variables, only the PBI was independently associated with mortality in the multivariate logistic regression analysis. Non-survivors had significantly higher PBI scores compared to survivors, highlighting that acute severity of illness at the time of bacteremia onset may be the principal determinant of bacteremia outcome. This is in accordance with other studies that also show an association of PBI and mortality in bacteremia by VRE or other bacteria [30]. In contrast, traditional baseline factors such as age, sex, comorbidities, polymicrobial infection, and primary versus secondary bacteremia were not significantly associated with mortality.

Although shorter duration of appropriate antimicrobial therapy and duration of hospitalization were negatively associated with survival, this likely reflects reverse causality, as patients who died earlier had less opportunity to complete prolonged treatment courses and stay in the hospital longer. For this reason, these parameters were not included in the multivariate model. Notably, appropriate empirical therapy was administered in fewer than half of the evaluable cases (41.7%), yet it was not statistically associated with improved survival. Interestingly, a large cross-sectional study of 183 hospitals in the USA showed that appropriate empirical therapy for VRE bacteremia was administered only in only 60.4% of cases [31]. The results of the same study that evaluated appropriateness of antimicrobial therapy in many different types of bacteremias, of whom VRE represented less than 1%, suggest that using appropriate initial empirical antimicrobial therapy can be associated with a lower inpatient hospital mortality. In the present study, the most commonly used inappropriate treatment was vancomycin in about half the patients, implying that the clinicians caring for these patients correctly suspected the possibility of infection by a Gram-positive microorganism but chose an antimicrobial whose spectrum did not cover VRE. Notably, the choice of empirical antimicrobial therapy may differ in different settings. Thus, clinicians practicing in Greece or other countries that have hospitals endemic in MDR pathogens, such as VRE, should considered using other antimicrobials for Gram-positive coverage, such as daptomycin or linezolid when *Enterococcus* infection is suspected. However, more studies should be performed to further describe the exact clinical scenarios where this should be warranted.

Survivors were significantly more likely to undergo follow-up blood cultures and to receive longer courses of appropriate therapy. While causality cannot be inferred, these findings suggest that closer microbiological monitoring and sustained targeted therapy may be markers of a more comprehensive clinical management [32]. Follow-up blood cultures are recommended in persistent bacteremia and high-risk infections; their lower frequency in non-survivors may reflect early death or differences in clinical decision-making [33,34].

Interestingly, in the post-COVID-19 era, antimicrobial regimens were more frequently adjusted according to susceptibility testing. This may indicate improved antimicrobial stewardship practices or heightened awareness of antimicrobial resistance patterns. However, this shift was not associated with reduced mortality.

The sustained increase in VRE bacteremia incidence combined with high mortality has significant implications. First, it highlights the urgent need to reinforce infection prevention and control measures despite the fact that in the ICUs of the study’s hospital, specific central line insertion and care bundles exist and audits show adequate implementation. Second, antimicrobial stewardship programs must continue to focus on reducing unnecessary glycopeptide and broad-spectrum antibiotic use, which may select for VRE [35]. The relatively low rate of appropriate empirical therapy reflects the challenge of balancing adequate early coverage against the risk of promoting resistance. Development of local risk stratification tools to identify patients at high risk for VRE bacteremia could optimize empirical choices [36]. To that end, routine active surveillance of rectal carriage can expedite the treatment for VRE infections. Several studies have evaluated the role of this routine active surveillance for early detection of high-risk patients colonized with VRE, allowing fewer VRE infections in total, lower costs, and better empirical coverage in the event of an infection in colonized patients [37,38]. Third, the strong association between PBI and mortality reinforces the need for early recognition and aggressive supportive management of septic patients. Rapid diagnostic tools and prompt infectious disease consultation may help improve timeliness of appropriate therapy and source control [39,40].

Several limitations of the present study should be acknowledged. The retrospective design introduces potential information bias and limits causal inference. The single-center setting may reduce generalizability to other institutions. The relatively small sample size limited statistical power and precluded robust multivariate modeling. Moreover, the small number in the pre-COVID study may be associated with a low statistical power, thus making the results potentially unstable. Additionally, the absence of molecular typing prevents conclusions regarding clonal spread or resistance mechanisms beyond phenotypic characterization.

## 5. Conclusions

To conclude, VRE bacteremia incidence increased dramatically over the six-year study period in the present single center study, with persistently high in-hospital mortality. Severity of illness at presentation, as measured by the PBI, was independently associated with in-hospital mortality. Despite some improvements in antimicrobial management practices in the post-COVID-19 era, mortality remained unchanged. These findings emphasize the urgent need for strengthened infection control strategies, optimized antimicrobial stewardship, and early aggressive management of critically ill patients to mitigate the growing impact of VRE bacteremia.

## Figures and Tables

**Table 1:** Characteristics of patients with VRE bacteremia in total and in regard to mortality.

Characteristic	All Patients (*n* = 96) *	Lived (*n* = 44)	Died (*n* = 52)	*p*-Value
Pre-COVID-19 era, *n* (%)	15 (15.6)	9 (20.5)	6 (11.5)	0.2684
Male, *n* (%)	54 (56.3)	28 (63.6)	26 (50)	0.2177
Age, years, mean (SD)	68.6 (14.5)	67.7 (15.6)	69.4 (13.6)	0.5732
Myocardial infarction, *n* (%)	14/91 (15.4)	6/42 (14.3)	8/49 (16.3)	1
Heart failure, *n* (%)	12/91 (13.2)	3/42 (7.1)	9/49 (18.4)	0.1336
Cerebrovascular disease, *n* (%)	8/91 (8.8)	5/42 (11.9)	3/49 (6.1)	0.4634
Chronic lung disease, *n* (%)	12/91 (13.2)	5/42 (11.9)	7/49 (14.3)	0.7675
Diabetes mellitus, *n* (%)	24/91 (26.4)	13/42 (31)	11/49 (22.4)	0.4748
Solid malignancy, *n* (%)	26/91 (28.6)	10/42 (23.8)	16/49 (32.7)	0.4855
Hematologic malignancy, *n* (%)	9/91 (9.9)	3/42 (7.2)	6/49 (12.2)	0.4977
Charlson comorbidity index, median (IQR)	5 (3–7)	4 (3–6)	5 (4–7)	0.0656
Central venous catheter, *n* (%)	78 (81.3)	33 (75)	45 (86.5)	0.1921
Recent hospitalization, *n* (%)	38/83 (45.8)	15/40 (37.5)	23/43 (53.5)	0.1871
Recent antimicrobial use, *n* (%)	29/68 (42.6)	11/33 (33.3)	18/35 (51.4)	0.1492

COVID-19: coronavirus disease 2019; IQR: interquartile range; SD: standard deviation; *: denominators are total number as described on top, unless otherwise mentioned.

**Table 2:** Characteristics of infection data of VRE bacteremia in total and in regard to mortality.

Characteristic	All Patients (*n* = 96) *	Lived (*n* = 44)	Died (*n* = 52)	*p*-Value
Pre-infection length of stay, days, median (IQR)	20 (9–33)	21 (9–35)	19.5 (9–32.5)	0.7734
Polymicrobial infection, *n* (%)	40 (41.7)	19 (43.2)	21 (40.4)	0.8371
Pitt bacteremia index, median (IQR)	2 (0–4)	0 (0–2)	2.5 (0–6)	0.0051
Ward at infection				
Medical, *n* (%)	45 (46.9)	21 (47.7)	24 (46.2)	1
ICU, *n* (%)	39 (40.6)	13 (29.5)	26 (50)	0.062
Surgical, *n* (%)	12 (12.5)	10 (22.7)	2 (3.8)	0.0105
Primary bacteremia, *n* (%)	54/95 (56.8)	26/43 (60.5)	28 (53.8)	0.5398
Follow-up blood culture, *n* (%)	81/94 (86.2)	42/43 (97.7)	39/51 (76.5)	0.0027
Appropriate empirical treatment, *n* (%)	35/84 (41.7)	13/36 (36.1)	22/48 (45.8)	0.5027
Antimicrobials changed based on susceptibility, *n* (%)	46/90 (51.1)	24/40 (60)	22/50 (44)	0.1440
Duration of appropriate treatment, days, median (IQR)	9 (4.8–14)	12 (7–15)	7 (3–11)	0.0115
Duration of hospitalization post infection, days, median (IQR)	18 (7–34)	25 (14–38)	10.5 (3–29.5)	0.0009
In-hospital mortality, *n* (%)	52 (54.2)	NA	NA	NA

ICU: intensive care unit; IQR: interquartile range; NA: not applicable; *: denominators are total number as described on top, unless otherwise mentioned.

**Table 3:** Characteristics of patients with VRE bacteremia per year of the study period.

Characteristic	2018 (*n* = 5) *	2019 (*n* = 10)	2020 (*n* = 10)	2021 (*n* = 22)	2022 (*n* = 28)	2023 (*n* = 21)
Patient-days	206,300	203,179	183,272	198,844	208,345	217,726
VRE bacteremia per 10,000 patient-days	0.242	0.492	0.546	1.106	1.344	1.001
Male, *n* (%)	4 (80)	6 (60)	7 (70)	9 (40.9)	18 (64.3)	10 (47.6)
Age, years, mean (SD)	61 (23.5)	61.6 (17.6)	71.2 (13.8)	71 (16)	68.9 (10.6)	69.4 (14)
Myocardial infarction, *n* (%)	0/4 (0)	1/7 (14.3)	0 (0)	3 (13.6)	8/27 (29.6)	2 (9.5)
Heart failure, *n* (%)	0/4 (0)	2/7 (28.6)	2 (20)	2 (9.1)	5/27 (18.5)	1 (4.8)
Cerebrovascular disease, *n* (%)	1/4 (25)	1/7 (14.3)	1 (10)	2 (9.1)	2/27 (7.4)	1 (4.8)
Chronic lung disease, *n* (%)	0/4 (0)	1/7 (14.3)	1 (10)	4 (18.2)	6/27 (22.2)	0 (0)
Diabetes mellitus, *n* (%)	2/4 (50)	2/7 (28.6)	0 (0)	5 (22.7)	11/27 (40.7)	4 (19)
Solid malignancy, *n* (%)	2/4 (50)	2/7 (28.6)	4 (40)	4 (18.2)	7/27 (25.9)	7 (33.3)
Hematologic malignancy, *n* (%)	0/4 (0)	0/7 (0)	1 (10)	1 (4.5)	4/27 (14.8)	3 (14.3)
Charlson comorbidity index, median (IQR)	4 (2–8.3)	5 (3–7)	5.5 (3.5–7.3)	4 (2.8–6)	6 (4–7)	5 (3.5–6.5)
Central venous catheter, *n* (%)	5 (100)	9 (90)	8 (80)	21 (95.5)	21 (75)	14 (66.7)
Recent hospitalization, *n* (%)	3/4 (75)	6/8 (75)	5/9 (55.6)	4/19 (21.1)	10/24 (41.7)	10/19 (52.6)
Recent antimicrobial use, *n* (%)	2/3 (66.7)	3/5 (60)	3/7 (42.9)	4/18 (22.2)	9/20 (45)	8/15 (53.3)

IQR: interquartile range; SD: standard deviation; VRE: vancomycin-resistant *Enterococcus*; *: denominators are total number as described on top, unless otherwise mentioned.

**Table 4:** Characteristics of infection data of VRE bacteremia per year of the study period.

Characteristic	2018 (*n* = 5) *	2019 (*n* = 10)	2020 (*n* = 10)	2021 (*n* = 22)	2022 (*n* = 28)	2023 (*n* = 21)
Pre-infection length of stay, days, median (IQR)	11.5 (9–19.3)	19.5 (10.5–46)	10.5 (1–27)	24.5 (12.5–34.8)	21.5 (10.5–37.3)	22 (7.5–35)
Polymicrobial infection, *n* (%)	3 (60)	3 (30)	6 (60)	6 (27.3)	11 (39.3)	11 (52.4)
Pitt bacteremia index, median (IQR)	1 (0–2)	1 (0–5)	0 (0–5)	3 (1.5–5.5)	0 (0–3)	0 (0–5)
Primary bacteremia, *n* (%)	1/4 (25)	7 (70)	8 (80)	12 (54.5)	14 (50)	12 (57.1)
Ward at infection						
Medical, *n* (%)	2 (40)	5 (50)	7 (70)	8 (36.4)	12 (42.9)	11 (52.4)
ICU, *n* (%)	2 (40)	3 (30)	3 (30)	10 (45.5)	12 (42.9)	9 (42.9)
Surgical, *n* (%)	1 (20)	2 (20)	0 (0)	4 (18.2)	4 (14.3)	1 (4.8)
Follow-up blood culture, *n* (%)	3/4 (75)	7 (70)	8 (80)	21/21 (100)	23 (82.1)	19 (90.5)
Appropriate empirical treatment, *n* (%)	2/2 (100)	4/9 (44.4)	2 (20)	6/19 (31.6)	11/24 (45.8)	10/20 (50)
Antimicrobials changed based on susceptibility, *n* (%)	0/2 (0)	2/9 (22.2)	6 (60)	13/21 (61.9)	14/27 (51.9)	11 (52.4)
Duration of appropriate treatment, days, median (IQR)	14 (14–14)	5 (5–13)	7.5 (2.5–11.8)	11 (4–13)	10 (3–15)	8.5 (6.3–13.8)
Duration of hospitalization post infection, days, median (IQR)	18 (8.3–22.5)	14.5 (5–34.8)	18 (1.8–41)	14.5 (8.5–39.3)	16.5 (8.3–28)	20 (3.5–49.5)
In-hospital mortality, *n* (%)	1 (20)	5 (50)	6 (60)	14 (63.6)	15 (53.6)	11 (52.4)

ICU: intensive care unit; IQR: interquartile range; VRE: vancomycin-resistant *Enterococcus*; *: denominators are total number as described on top, unless otherwise mentioned.

**Table 5:** Multivariate logistic regression analysis of mortality in patients with bacteremia by vancomycin-resistant *Enterococcus*.

Characteristic	Multivariate Analysis *p*-Value	OR (95% CI)
Charlson comorbidity index, per point	0.070	1.193 (0.986–1.442)
Pitt bacteremia index, per point	0.001	1.449 (1.166–1.801)
Surgical ward at diagnosis	0.581	1.475 (0.371–5.870)

CI: confidence interval; OR: odds ratio.

## Data Availability

Data are available upon reasonable request from the corresponding author.

## Supplementary Materials

The following supporting information can be downloaded at: https://www.mdpi.com/article/10.3390/pathogens15050467/s1. Table S1: Characteristics of patients with VRE bacteremia before and after the COVID-19 pandemic; Table S2: Characteristics of infection data of VRE bacteremia before and after the COVID-19 pandemic.
