# Supplementary material for: Temporal Trends and Mortality of Vancomycin-Resistant Enterococcus Bacteremia—A Six-Year Retrospective Cohort Study in a Tertiary Hospital in Greece
Source: Pathogens. 2026 Apr 25;15(5):467. doi: 10.3390/pathogens15050467 (PMC13209235; doi:10.3390/pathogens15050467)
Supplement: Supplementary file 1 [file pathogens-15-00467-s001.zip › pathogens-4264436-supplementary.pdf]

Table S1. Characteristics of patients with VRE bacteremia before and after the COVID-19 pandemic.

Characteristic	Pre-COVID-19 (*n* = 15)	Post-COVID-19 (*n* = 81)	p-value
Male, *n* (%)	10 (66.6)	44 (54.3)	0.4124
Age, years, mean (SD)	61.4 (18.9)	69.9 (13.3)	0.0365
Myocardial infarction, *n* (%)	1 (6.6)	13/80 (16.3)	1
Heart failure, *n* (%)	2 (13.3)	10/80 (12.5)	0.6347
Cerebrovascular disease, *n* (%)	2 (13.3)	6/74 (7.5)	0.2477
Chronic lung disease, *n* (%)	1 (6.6)	11/80 (13.8)	1
Diabetes mellitus, *n* (%)	4 (26.6)	20/80 (25)	0.4717
Solid malignancy, *n* (%)	4 (26.6)	22/80 (27.5)	0.7227
Hematologic malignancy, *n* (%)	0/11 (0)	9/80 (11.3)	0.5932
Charlson comorbidity index, median (IQR)	5 (2-7)	5 (3-7)	0.9215
Central venous catheter, *n* (%)	14 (93.3)	64 (79)	0.2892
Recent hospitalization, *n* (%)	9/12 (75)	29/71 (40.8)	0.0567
Recent antimicrobial use, *n* (%)	5/8 (62.5)	24/60 (40)	0.2719

IQR: interquartile range; NA: not applicable; SD: standard deviation; VRE: vancomycin-resistant *Enterococcus*; *: denominators are total number as described on top, unless otherwise mentioned.

Table S2. Characteristics of infection data of VRE bacteremia before and after the COVID-19 pandemic.

Characteristic	Pre-COVID-19 (*n* = 15)	Post-COVID-19 (*n* = 81)	p-value
Pre-infection length of stay, days, median (IQR)	16 (9-31)	21 (8.5-34)	0.8873
Polymicrobial infection, *n* (%)	6 (40)	34 (42)	1
Pitt bacteremia index, median (IQR)	1 (0-4)	2 (0-4.3)	0.6891
Ward at infection			
Medical, *n* (%)	7 (46.7)	38 (46.9)	1
ICU, *n* (%)	5 (33.3)	34 (42)	0.5813
Surgical, *n* (%)	3 (20)	9 (11.1)	0.3931
Follow-up blood culture, *n* (%)	10/14 (71.4)	71/80 (88.8)	0.0999
Appropriate empirical treatment, *n* (%)	6/11 (54.5)	29/73 (39.7)	0.5134
Antimicrobials changed based on susceptibility, *n* (%)	2/11 (18.2)	44/79 (55.7)	0.0141
Duration of appropriate treatment, days, median (IQR)	7 (5-13.8)	9.5 (4-14)	0.8293
Duration of hospitalization post infection, days, median (IQR)	35.5 (21.3-72.8)	39 (21.5-68)	0.8253
In-hospital mortality, *n* (%)	6 (40)	46 (56.8)	0.2684

ICU: intensive care unit; IQR: interquartile range; VRE: vancomycin-resistant *Enterococcus*; *: denominators are total number as described on top, unless otherwise mentioned.
